# Suitability of Endobronchial Ultrasound-Guided Transbronchial Needle Aspiration versus Paired Transbronchial Biopsy Specimens for Evaluating Programmed Death Ligand-1 Expression in Stage III and IV Lung Cancer: A Comparative Retrospective Study

**DOI:** 10.7150/jca.55738

**Published:** 2021-05-27

**Authors:** Hiroki Matsuoka, Tomoyuki Araya, Toshiyuki Kita, Nanao Terada, Kenta Yamamura, Shingo Nishikawa, Yuichi Tambo, Takashi Sone, Hideharu Kimura, Akishi Ooi, Satomi Kasashima, Atsuhiro Kawashima, Kazuo Kasahara

**Affiliations:** 1 Department of Respiratory Medicine, Faculty of Medicine, Institute of Medical, Pharmaceutical and Health Sciences, Kanazawa University.; 2 Department of Respiratory Medicine, Kanazawa University Hospital.; 3 Department of Respiratory Medicine, National Hospital Organization Kanazawa Medical Center.; 4 Department of Molecular and Cellular Pathology, Graduate School of Medical Science, Kanazawa University.; 5 Department of Clinical Laboratory Science, Faculty of Health Sciences, Kanazawa University.; 6 Department of Pathology, National Hospital Organization Kanazawa Medical Center.

**Keywords:** programmed death ligand-1, endobronchial ultrasound-guided transbronchial needle aspiration, non-small cell lung cancer, small cell lung cancer, immune checkpoint inhibitor.

## Abstract

**Objectives:** Cancer cells usually escape tumor-reactive T-cell responses using immune checkpoint proteins, such as programmed death protein-1 (PD-1) and its ligand, programmed death ligand-1 (PD-L1). These proteins can be blocked by immune checkpoint inhibitors (ICIs); the decision on ICI-based first-line treatment for advanced lung cancers depends on the PD-L1 levels in tumor specimens. Determining the PD-L1 expression conventionally requires histological specimens from resected tumors and core biopsy specimens. Non-small cell lung cancer (NSCLC) is usually diagnosed at stage III or IV; therefore, only small biopsy specimens, such as those obtained via endobronchial ultrasound-guided transbronchial needle aspiration (EBUS-TBNA) are available. However, the suitability of EBUS-TBNA specimens determining the PD-L1 expression levels in advanced lung cancers remains unclear.

**Materials and Methods:** Here, we investigated the concordance rate of PD-L1 expression between EBUS-TBNA and matched transbronchial biopsy (TBB) specimens. Using the 22C3 anti-PD-L1 antibody (immunohistochemistry), we determined the PD-L1 expression levels in paired specimens obtained from 69 patients (50 with advanced NSCLC and 19 with small cell lung cancer [SCLC]), as well as the efficacy of ICIs in these patients.

**Results:** The concordance rate of PD-L1 expression between the EBUS-TBNA and TBB specimens was 78.3%. The κ values referent to the PD-L1-positive expression rate between EBUS-TBNA and TBB specimens were 0.707 and 0.676 at cutoff limits of ≥1% and ≥50%, respectively. Among the 19 SCLC patients, 16 (84.2%) exhibited no PD-L1 expression in both EBUS-TBNA and TBB specimens. Notably, the progression-free survival of patients with ≥50% PD-L1 expression in the paired specimens who received ICI treatment was 8.3 months.

**Conclusion:** Collectively, our results validate the use of EBUS-TBNA specimens for the determination of the PD-L1 expression levels in the context of NSCLC and SCLC.

## Introduction

Immune checkpoint inhibitors (ICIs) block immune checkpoint proteins such as programmed death protein-1 (PD-1) and its ligand, programmed death ligand-1 (PD-L1), thereby preventing the innate cytotoxic T-cell response against non-small cell lung cancer (NSCLC) cells 1. Clinical trials of ICIs such as PD-1/PD-L1 inhibitors (nivolumab, pembrolizumab, and atezolizumab) have demonstrated a positive association between the degree of ICI efficacy and the PD-L1 expression levels as per immunohistochemistry in both chemo-naïve and chemotherapeutically treated advanced NSCLC patients 2-5. Therefore, first-line treatment decisions for advanced NSCLC currently depend on the PD-L1 expression status in tumor specimens. The determination of the expression of PD-L1 conventionally requires histological specimens from resected tumors or core biopsies, rather than those obtained using endobronchial ultrasound-guided transbronchial needle aspiration (EBUS-TBNA) 3, 4. However, in the clinical practice, more than half of the NSCLC patients are diagnosed with locally advanced (stage III) or metastatic (stage IV) disease, and, therefore, only small biopsy specimens such as those obtained via EBUS-TBNA are available. Of note, EBUS-TBNA specimens perform equivalently to those obtained histologically for the diagnosis and staging of NSCLC 6, 7. However, although the use of EBUS-TBNA specimens for the molecular analysis of epidermal growth factor receptor (EGFR) and anaplastic large-cell lymphoma kinase (ALK) is well documented 8, 9, only a few studies have examined their suitability for the determination of the PD-L1 expression levels 10-18. Of note, these studies were based on a small number of specimens and did not use the 22C3 anti-PD-L1 antibody, which is widely used for the support of ICI-based treatment decisions (such as those in the context of pembrolizumab treatment) 3, 4, 19-21. SCLC patients have a low positive rate of PD-L1 expression 22-27, and little is known about the concordance rate of the PD-L1 expression between EBUS-TBNA and matched TBB specimens obtained from the same patient, in the context of both SCLC and NSCLC. Therefore, confirming the suitability of EBUS-TBNA specimens for PD-L1-based ICI treatment will both impact patient outcomes and avoid the negative effects associated with invasive diagnostics and ineffective therapies. Thus, in this comparative retrospective study, we determined the concordance rate of PD-L1 expression between EBUS-TBNA and matched TBB specimens obtained from the same patient (with stage III/IV NSCLC or SCLC) using the 22C3 anti-PD-L1 antibody. Additionally, we also clarified the therapeutic efficacy of ICIs based on the PD-L1 expression levels.

## Materials and Methods

### Ethics Statement

The present study was approved by the ethics committees of the Kanazawa University Hospital (#2018-061) and the National Hospital Organization Kanazawa Medical Center (#H30-064). Written informed consent was obtained from all patients. The study was performed in accordance with the ethical standards established by the Declaration of Helsinki.

### Patients

**Figure 1:**
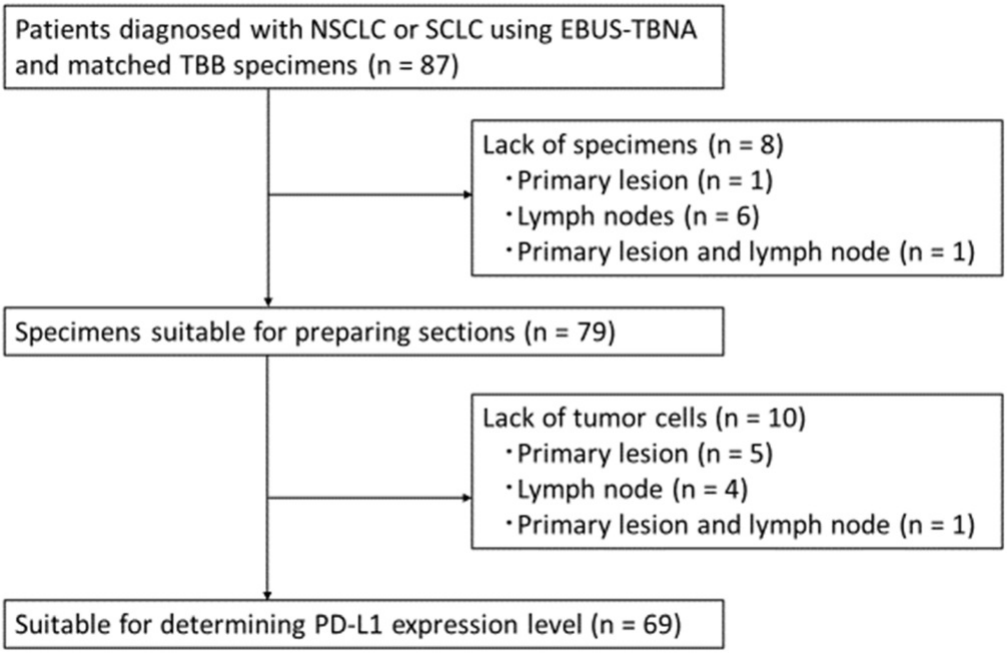
CONSORT flow diagram. Abbreviations: NSCLC: non-small cell lung cancer; SCLC: small cell lung cancer; EBUS-TBNA: endobronchial ultrasound-guided transbronchial needle aspiration; TBB: transbronchial biopsy; PD-L1: programmed death ligand-1.

A total of 87 consecutive patients diagnosed with NSCLC or SCLC using EBUS-TBNA and matched TBB specimens were screened for enrollment between April 2013 and March 2020. The patients were enrolled according to the following eligibility criteria: i) age ≥20; ii) histologically diagnosed lung cancer; iii) TBB and EBUS-TBNA were concurrently performed and tumor specimens were obtained in the context of both procedures; and iv) each specimen contained at least 100 cancer cells. The screening identified 18 patients with no residual tumor in their blocks; the remaining 69 patients showed paired specimens of EBUS-TBNA and TBB suitable for the determination of the PD-L1 expression levels (Figure 1). Data on the patients' characteristics were collected from the electronic medical records.

### Specimens: collection and processing

Bronchoscopy was performed according to the CHEST guidelines 9 prior to the study. Briefly, a dedicated flexible bronchoscope (BF-UC260FW; Olympus, Tokyo, Japan) equipped with an endoscopic ultrasound processor (EU-ME2; Olympus, Tokyo, Japan) was used to perform EBUS-TBNA. First, TBB specimens were obtained using the EBUS equipment with a guide sheath method (EBUS-GS) and disposable biopsy forceps (FB-231D; Olympus, Tokyo, Japan) 28. Thereafter, EBUS-TBNA was performed using a 22-gauge needle (NA-U401SX-4022; Olympus, Tokyo, Japan). The tissue specimens collected by performing EBUS-TBNA and TBB were immediately fixed in 10% neutral buffered formalin for 24 h and embedded in paraffin. The tissue blocks were then sliced into 4‒5-μm-thick sections at the time of diagnosis and used for the determination of the expression of PD-L1 expression.

### Immunohistochemistry

Immunohistochemistry (IHC) was performed to determine the PD-L1 expression levels using a mouse monoclonal anti-human PD-L1 antibody (clone 22C3 PharmDx, Dako/Agilent, Glostrup, Denmark) and the Dako Autostainer Link 48 system (Dako) in the context of an automated staining protocol 29. Of note, we used this specific antibody clone since the PD-L1 IHC 22C3 companion diagnostic assay is being used across institutions globally for the determination of PD-L1 expression in pembrolizumab-treated NSCLC patients. Importantly, the PD-L1 expression was determined only in tumor cells within EBUS-TBNA and TBB specimens, but not in tumor-associated macrophages/immune cells. Two experienced pathologists, blinded to the clinical data, independently evaluated all the slides. At least 100 viable tumor cells were required to determine PD-L1 IHC positivity on a slide 21, 30. A tumor cell was considered positive for PD-L1 staining if its membrane was partially or completely stained for the protein. In contrast, cytoplasm-stained cells were not considered positively stained. The tumor proportion score (TPS) was defined as the percentage of positive tumor cells among all tumor cells evaluated. We used two PD-L1 IHC cutoff limits (≥1% and ≥50%) for the determination of tumor cell positivity and, therefore, classified the PD-L1 expression rate into the following three categories: TPS <1%, TPS = 1‒49%, and TPS ≥50.

### Statistical analysis

The concordance rate of the PD-L1 expression levels between EBUS-TBNA and matched TBB specimens (considering both cutoff limits) was determined using κ statistics. The κ coefficient of concordance was calculated using the Pearson's test. The level of concordance was classified as poor (κ <0.00), slight (κ = 0.00‒0.20), fair (κ = 0.21‒0.40), moderate (κ = 0.41‒0.60), substantial (κ = 0.61‒0.80), and almost perfect (κ = 0.81‒1.00), as reported elsewhere 31. Tumor response was assessed based on the Response Evaluation Criteria in Solid Tumors (RECIST), version 1.1 32. Progression-free survival (PFS) was assessed by the Kaplan-Meier method; differences between groups were defined using the log-rank test. A p-value of <0.05 was considered statistically significant. All statistical analyses were performed using EZR (Saitama Medical Center, Jichi Medical University; Kanda, Japan, 2012), the graphical user interface for R (The R Foundation for Statistical Computing, Vienna, Austria, version 2.13.0).

## Results

### Patient characteristics

The clinical characteristics of the 69 patients from which paired specimens were obtained via EBUS-TBNA and TBB for the assessment of tumor PD-L1 expression are summarized in Table 1.

### Immunohistochemistry staining of PD-L1

**Figure 2:**
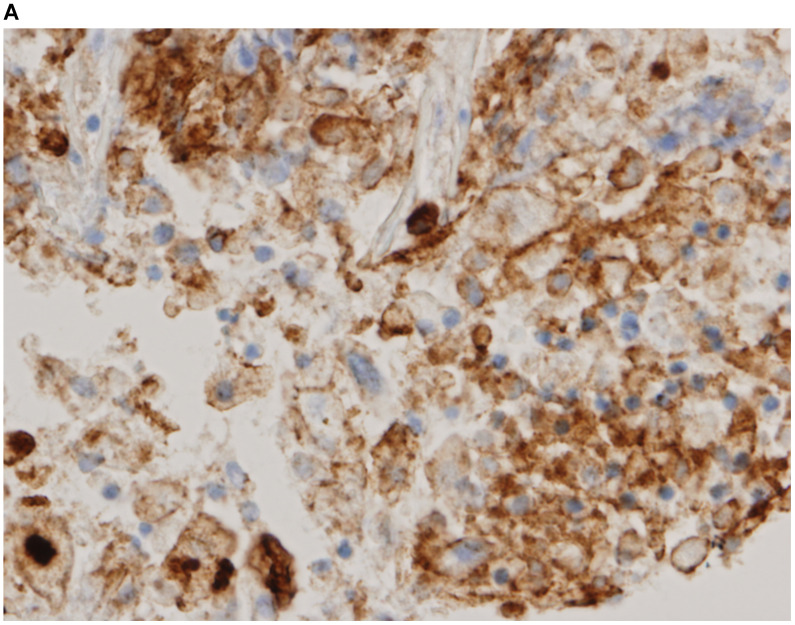

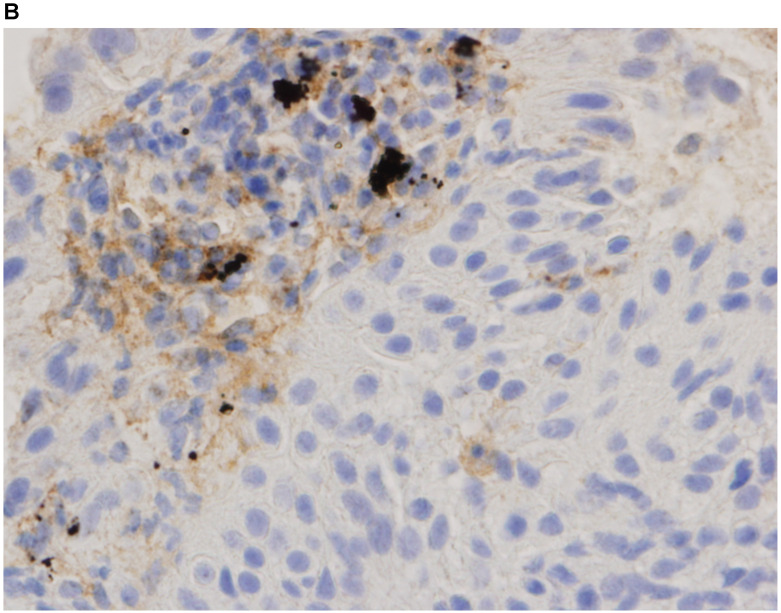

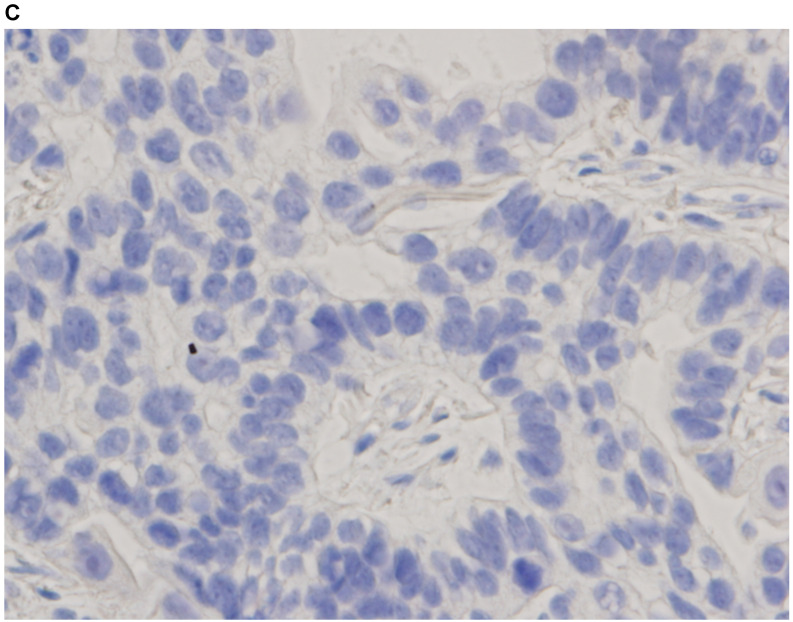

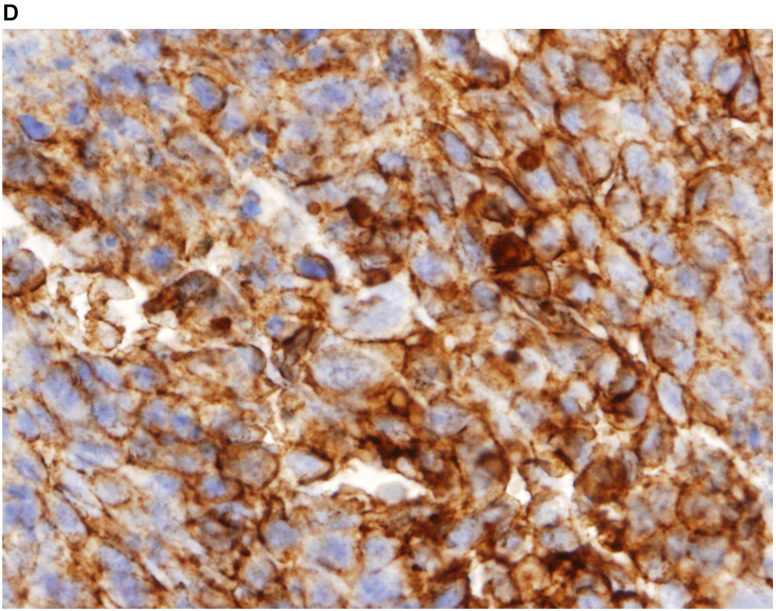

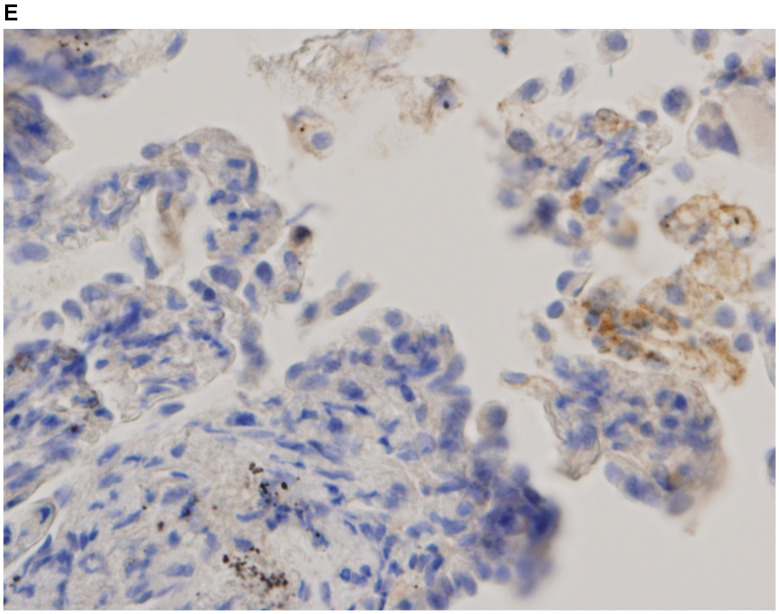

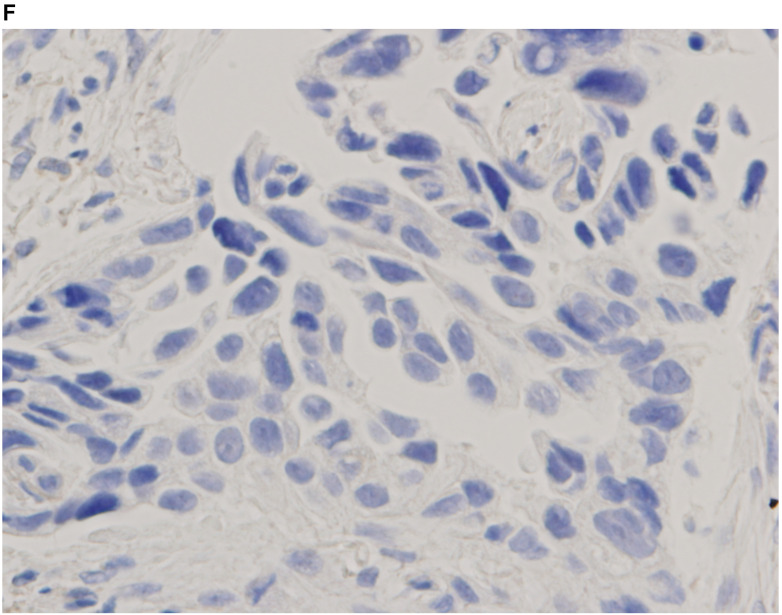
Immunohistochemistry characterization. Representative images of programmed death ligand-1 immunohistochemistry staining of tumor cells obtained via endobronchial ultrasound-guided transbronchial needle aspiration (**A** tumor proportion score [TPS] ≥50%, **B** TPS = 1‒49%, **C** TPS <1%) and transbronchial biopsy (**D** TPS ≥50%, **E** TPS = 1‒49%, **F** TPS <1%) (40x magnification).

Representative images of PD-L1 immunohistochemistry staining of tumor cells obtained via EBUS-TBNA and TBB are shown in Figure 2.

### Concordance of PD-L1 expression

**Figure 3:**
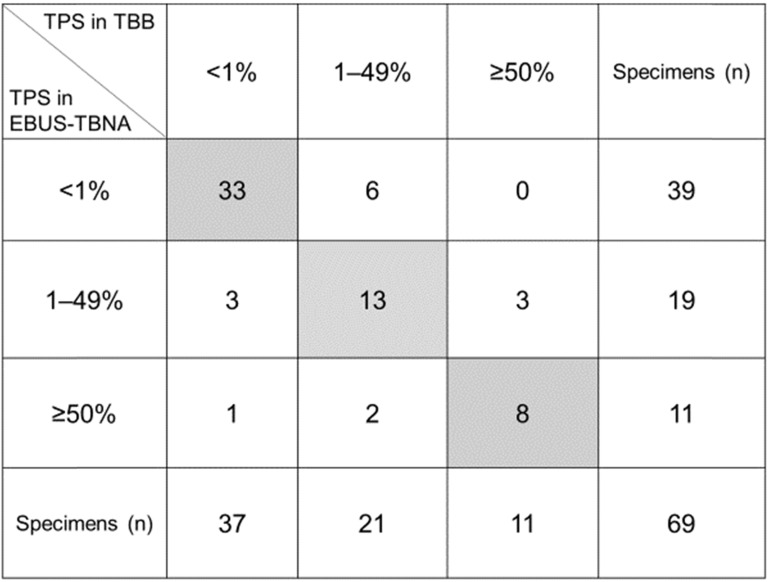
Comparison of the expression levels of programmed death ligand-1 (PD-L1) in endobronchial ultrasound-guided transbronchial needle aspiration (EBUS-TBNA) versus matched transbronchial biopsy (TBB) specimens. The concordance rate of PD-L1 expression between EBUS-TBNA and matched TBB specimens was 78.2% (54/69, the sum is given in the gray box). The κ values of the PD-L1-positive expression rate between EBUS-TBNA and matched TBB specimens were 0.707 and 0.676 for the cutoff limits of ≥1% and ≥50%, respectively. Abbreviations: TPS: tumor proportion score; EBUS-TBNA: endobronchial ultrasound-guided transbronchial needle aspiration; TBB: transbronchial biopsy.

The comparison of the PD-L1 expression levels between EBUS-TBNA and matched TBB specimens is shown in Figure 3. Category-wise, the distribution of the 69 EBUS-TBNA specimens was as follows: 39 (56.5%), TPS <1%; 19 (27.5%), TPS = 1‒49%; and 11 (16.0%), TPS ≥50%, whereas that of the 69 TBB specimens was as follows: 37 (53.6%), TPS <1%; 21 (30.4%), TPS = 1‒49%; and 11 (16.0%), TPS ≥50%. Of note, the concordance rate of the expression of PD-L1 between EBUS-TBNA and matched TBB specimens was 78.3% (54/69) as shown in Figure 3. The remaining 15 cases (21.7%) showed discordance between EBUS-TBNA and matched TBB specimens. Among these 15 patients, only one showed high PD-L1 expression levels in the EBUS-TBNA, but not in the TBB specimen. Moreover, the κ values of the PD-L1-positive expression rate between EBUS-TBNA and matched TBB specimens were 0.707 (substantial) and 0.676 (substantial) considering the cutoff limits of ≥1% and ≥50%, respectively.

### PD-L1 in NSCLC and SCLC

**Figure 4:**
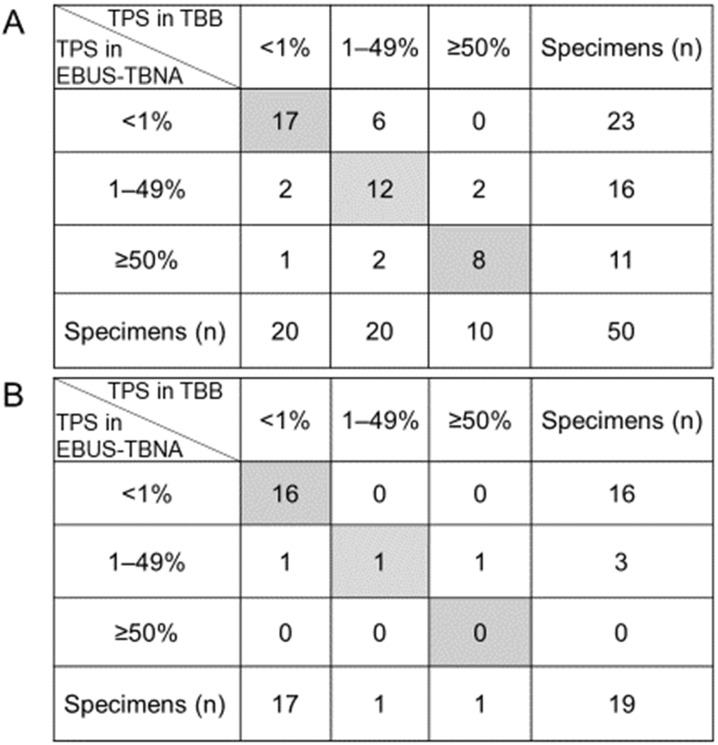
Differences in the expression of programmed death ligand-1 (PD-L1) between non-small cell lung cancer (NSCLC) and small cell lung cancer (SCLC). (A) Comparison of the PD-L1 expression levels between EBUS-TBNA and matched TBB specimens from NSCLC patients. The concordance rate of the PD-L1 expression between EBUS-TBNA and matched TBB specimens was 74.0% (37/50, the sum is given in the grey box). The κ values of the PD-L1-positive expression rate between EBUS-TBNA and matched TBB specimens were 0.634 and 0.699 for the cutoff limits of ≥1% and ≥50%, respectively. (B) Comparison of the PD-L1 expression levels between EBUS-TBNA and matched TBB specimens obtained from SCLC patients. The concordance rate was 89.5% (17/19, the sum is given in the gray box). The κ value of the PD-L1-positive expression rate was 0.771 for the cutoff limit of ≥1%. The κ value for the cutoff limit of ≥50% could not be calculated because only one SCLC patient showed high PD-L1 expression in either EBUS-TBNA or TBB specimens. Abbreviations: TPS: tumor proportion score; EBUS-TBNA: endobronchial ultrasound-guided transbronchial needle aspiration; TBB: transbronchial biopsy.

Previous studies demonstrated a good concordance of the PD-L1 expression levels between EBUS-TBNA and matched histological specimens, only in the context of NSCLC patients 10, 12-15, 17, 18. Here, we separately evaluated the TPS scores based on the histological type in the context of 50 NSCLC and 19 SCLC patients. The classification of the PD-L1 expression rate in the 50 EBUS-TBNA specimens obtained from NSCLC patients according to the three categories is depicted in Figure 4A. The concordance rate of PD-L1 expression between EBUS-TBNA and matched TBB specimens obtained from NSCLC patients was 74.0% (37/50). The κ values of the PD-L1-positive expression rate between EBUS-TBNA and matched TBB specimens in the context of NSCLC patients were 0.634 (substantial) and 0.699 (substantial) for cutoff limits of ≥1% and ≥50%, respectively. Additionally, the classification of the PD-L1 expression rate in 19 EBUS-TBNA specimens obtained from SCLC patients according to the three categories is depicted in Figure 4B. The concordance rate of the PD-L1 expression between EBUS-TBNA and matched TBB specimens obtained from SCLC patients was 89.5% (17/19). Additionally, the κ value of the PD-L1-positive expression rate was 0.771 (substantial) for the cutoff limit of ≥1%; the κ value for the cutoff limit of ≥50% could not be calculated because only one SCLC patient showed a high PD-L1 expression in either the EBUS-TBNA or TBB specimens. Importantly, the rates of positive PD-L1 expression (TPS ≥1%) in both EBUS-TBNA and matched TBB specimens obtained from NSCLC patients were significantly higher than those obtained from SCLC specimens (48.0% vs. 10.5%, *p* = 0.006) as shown in Figure 4A and 4B.

### Determining the efficacy of ICIs

**Figure 5:**
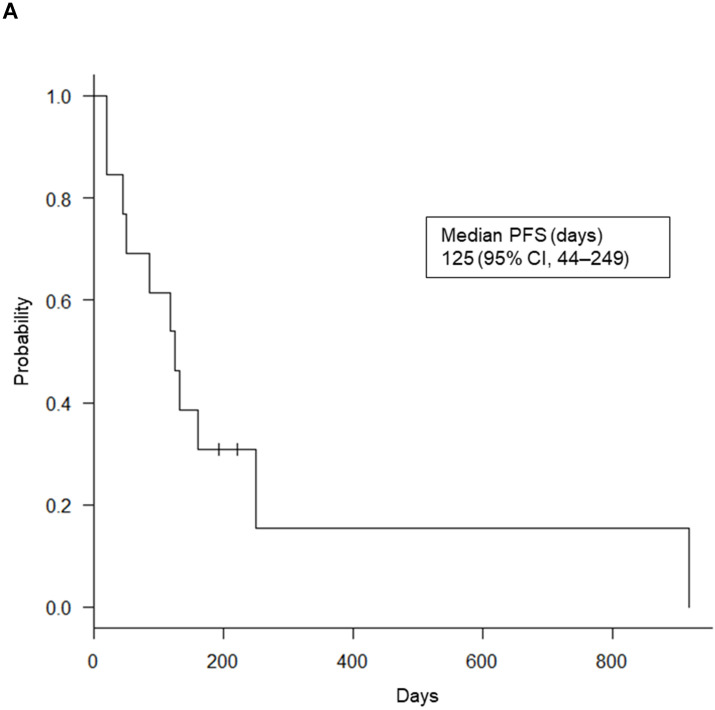

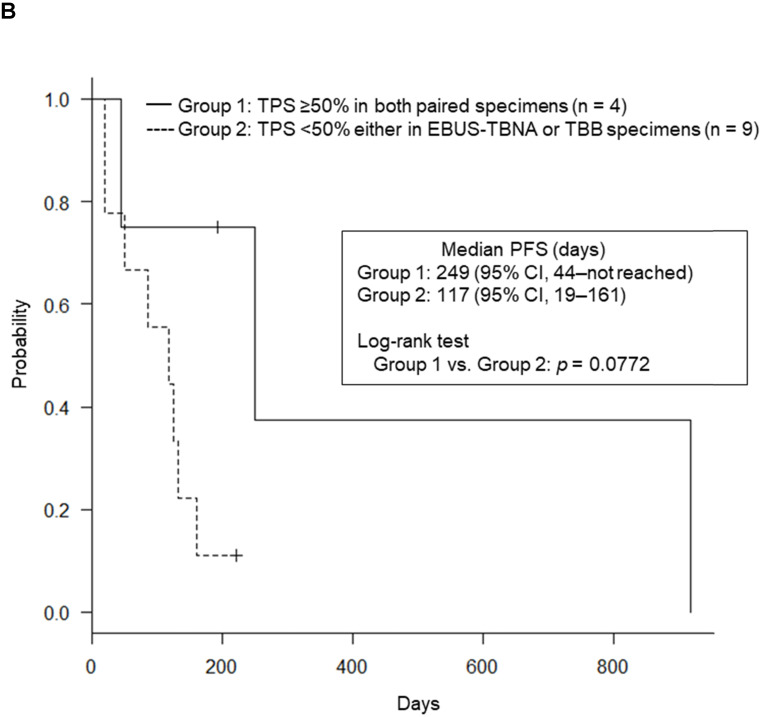
Progression-free survival (PFS) Kaplan-Meier curves. (A) For the 13 immune checkpoint inhibitors (ICIs)-treated patients. (B) For the four patients with high programmed death ligand-1 (PD-L1) expression both in endobronchial ultrasound-guided transbronchial needle aspiration (EBUS-TBNA) and matched transbronchial biopsy (TBB) specimens (Group 1), and for the remaining nine ICI-treated patients (Group 2). Abbreviation: CI: confidence interval; TPS: tumor proportion score.

Of the 69 patients, only 17 NSCLC patients (24.6%) received ICI treatment: nivolumab (n = 7), pembrolizumab (n = 8), and atezolizumab (n = 2). The median PFS for these 17 patients was 125 days (95% confidence interval [CI], 44-249). Six of the 17 patients showed high PD-L1 expression levels in both EBUS-TBNA and matched TBB specimens and a median PFS slightly higher than that of the remaining 11 patients (164 days vs. 117 days, *p* = 0.315). Of note, previously, the CheckMate017 study reported no correlation between the efficacy of nivolumab and the PD-L1 expression levels in patients with squamous cell carcinoma 33. Therefore, to analyze the survival data in a relevant population, we excluded the survival data of four patients with squamous cell carcinoma treated with nivolumab. In the remaining 13 ICI-treated NSCLC patients, we determined the relationship between PFS after ICI treatment and the PD-L1 expression levels in both EBUS-TBNA and matched TBB specimens, as shown in Table 2. Interestingly, among the 13 patients who received ICI treatment, 5 (38.4%) showed a partial response, and 4 (30.8%) showed stable disease (overall response rate, 38.4%; disease control rate, 69.2%). The remaining 4 patients (30.8%) showed progressive disease. The Kaplan-Meier curves of PFS for the 13 patients are shown in Figure 5A. The average median PFS was 125 days (95% CI, 44-249). Importantly, the median PFS of the four patients with high PD-L1 expression levels in both EBUS-TBNA and matched TBB specimens was higher than that in the remaining nine patients (249 days vs. 117 days, *p* = 0.0772), as shown in Table 2 and Figure 5B. Additionally, the median PFS based on the PD-L1 expression levels was also calculated. The median PFS for patients with concordant PD-L1 expression in both EBUS-TBNA and matched TBB specimens was 249 days (95% CI, 44 to not reached) in the TPS ≥50% category, 50 days (95% CI, 20 to not reached) in the TPS = 1‒49% category, and 131 days (95% CI, 117 to not reached) in the TPS <1% category, as shown in Table 2. On the other hand, the median PFS for patients with discordant PD-L1 expression between EBUS-TBNA and matched TBB specimens was 85 days (95% CI, 19 to not reached) as shown in Table 2.

## Discussion

Here, we demonstrate a high concordance in the PD-L1 expression levels between EBUS-TBNA and matched TBB samples obtained from patients with NSCLC and SCLC. We used the 22C3 clone, a widely used anti-PD-L1 antibody, to support the ICI-based treatment decisions. Our findings suggest that EBUS-TBNA specimens can be used as surrogate tissues for determining the PD-L1 expression when tissues from primary lesions are unavailable. Furthermore, we highlight the high efficacy of ICI in patients with high PD-L1 expression (concordant in paired specimens), in line with the results of a previous trial based on histological specimens other than those collected via EBUS-TBNA 4.

Table 3 summarizes the results of previous studies on the correlation of the PD-L1 expression levels between specimens obtained via EBUS-TBNA and those obtained via resection, TBB, computed tomography-guided biopsy, or ultrasound-guided core needle biopsy. Kitazono et al 10 reported a good correlation, whereas Ilie et al 11 reported a poor correlation between the PD-L1 expression levels in surgically resected specimens and matched small biopsy specimens, including TBNA specimens. However, these studies included only 12 TBNA specimens and did not use the 22C3 clone as the anti-PD-L1 antibody. In addition, their study populations mainly comprised patients with resectable stage I‒III NSCLC. Therefore, their results cannot be extrapolated to PD-L1 expression determined in EBUS-TBNA specimens obtained from advanced NSCLC patients. Sakakibara et al 12 also reported a strong correlation of the PD-L1 expression levels between EBUS-TBNA and matched specimens. However, they analyzed a small number of matched specimens (16 TBB, six resected lung cancer tissue, and five resected lymph node specimens), and did not provide information about the disease stage. In addition, they used rabbit monoclonal anti-PD-L1 antibody (clone EPR1161) instead of the 22C3 clone. Of note, four of the five studies 13, 15-17 that determined the PD-L1 expression levels using the 22C3 clone had no or a small number of matched specimens for comparison with EBUS-TBNA specimens (Table 3). Sakata et al 14 demonstrated that the PD-L1 expression in EBUS-TBNA specimens strongly correlated with that in resected tumor specimens at a PD-L1 cutoff of ≥1% and found a significant decrease in the sensitivity and positive predictive values of EBUS-TBNA specimens compared with those of resected tumors at a PD-L1 cutoff of ≥50%. Although their study used 61 pairs of EBUS-TBNA and resected lung specimens, the study population mainly consisted of patients with resectable stage-III NSCLC and included only one patient with stage IV NSCLC. Thus, these results cannot be directly translated into the daily practice given the greater utility of small biopsy specimens for determining the PD-L1 expression. Importantly, our results are consistent with those obtained by Yoshimura et al 18 who showed a good concordance rate of the PD-L1 expression between EBUS-TBNA and matched TBB specimens mainly from patients with advanced NSCLC. However, they also used the clone E1L3N and not the clone 22C3 as the anti-PD-L1 antibody. Thus, with advantages of including a comparatively larger number of matched EBUS-TBNA and TBB specimens and using the clone 22C3, our study clearly validated the utility of EBUS-TBNA specimens for determining the PD-L1 expression levels in NSCLC. Our study outcomes may be particularly useful as small biopsy specimens such as those obtained via EBUS-TBNA may be the only specimens available in the clinical practice, especially in the context of patients with advanced-stage lung cancer.

Furthermore, the previous studies rarely reported the ICI efficacy in association with the PD-L1 expression in context of EBUS-TBNA and matched TBB specimens (Table 3). Using 22C3, Perrotta et al 16 demonstrated that the PD-L1 expression levels were not affected by the tissue sampling method and that all patients responding to ICI treatment showed high PD-L1 expression levels. However, they used a variety of specimens in the absence of matched specimens to assess the PD-L1 levels, and therefore, the study was non-comparative 16.

In the present study, 15 patients showed discordant PD-L1 expression levels in the paired specimens. We attribute this result to the intra-tumoral heterogeneity of PD-L1 expression. Only one patient showed high PD-L1 expression levels in the EBUS-TBNA, but not in the TBB specimen. It is unclear whether the PD-L1 levels detected in the EBUS-TBNA specimen were a false positive or those in the TBB specimen a false negative, since the patient did not receive ICI treatment.

Various factors such as the limited number of patients and the intra-tumoral heterogeneity may have affected the PFS results. Nevertheless, we found a positive correlation between high PD-L1 expression levels in paired specimens and a long PFS, in line with previous studies 4, 34. The median PFS for patients without PD-L1 expression in both specimens was 131 days (4.4 months) in the present study, which is comparable to the PFS of 4.1 months in patients treated with docetaxel in the KEYNOTE-010 trial 19.

We observed negligible PD-L1 expression in SCLC specimens. However, the concordance rate between matched specimens from SCLC patients was 89.5%, higher than that in the context of specimens from NSCLC patients (74.0%). Previous studies using the anti-PD-L1 clones 28-8, E1L3N, SP263, and 22C3 showed relatively low PD-L1 expression levels in SCLC specimens (5.0‒25.0%) 22-27 compared with those in NSCLC specimens (66.0%) 19. Bonanno et al 22 analyzed the PD-L1 expression levels in 104 SCLC patients using the 22C3 clone and reported that the number of PD-L1-positive cases was significantly higher in stage I‒III *versus* metastatic patients (32% vs. 13%). Similarly, Ishii et al 35 reported that the expression of PD-L1 significantly correlated with the disease stage (i.e., stage I‒III SCLC). Together, these two studies highlighted that PD-L1 expression in SCLC patients is significantly higher during the early-stage disease, which is consistent with the results of the present study. Our results, in context of SCLC specimens, were also similar to those obtained with prospectively collected specimens of the CheckMate 032 SCLC cohort 27. EBUS-TBNA provides a high diagnostic yield in the context of SCLC and should, therefore, be preferentially recommended for patients with suspected SCLC 36, 37.

Although the PD-L1 expression levels in SCLC tumor tissues are yet to be recognized as a predictive marker for ICI 38, we believe that such a biomarker together or not with genomic mutations in SCLC tissues could be primarily evaluated using EBUS-TBNA specimens, which are easier to collect. However, more samples will be required from both NSCLC and SCLC patients for further genetic testing.

This study is not without limitations. First, as the study was retrospective and included a small sample size, the statistical power may be limited. Second, data pertaining to the PD-L1 expression levels just before the initiation of ICI treatment were not available since we used archived tissues obtained at the time of diagnosis. Third, we could not determine the intra-tumoral heterogeneity of PD-L1 expression because surgical specimens were unavailable. Fourth, the number of ICI-treated patients was low because ICI was not approved for treatment at the time of enrollment. Furthermore, the timing of ICI treatment and choice of prior regimens were based on the discretion of attending doctors, and therefore, were not standardized.

In conclusion, here we demonstrate a substantial concordance rate (78.3%) of the expression of PD-L1 (detected using the 22C3 clone) between EBUS-TBNA and matched TBB specimens collected from advanced lung cancer patients, and that the PD-L1 expression levels correlate with the clinical efficacy of ICIs in the treatment of NSCLC. Therefore, these findings validate the use of EBUS-TBNA specimens for the determination of the PD-L1 expression levels in NSCLC and SCLC.

## Figures and Tables

**Table 1:** Patient characteristics.

Characteristics (n = 69)		All patients, n (%)
Age	Median	72
	Range	38-83
Sex	Male	43 (62.3)
	Female	26 (37.7)
Smoking status	Current	23 (33.3)
	Former	30 (43.5)
	Never	16 (23.2)
Stage	II	1 (1.4)
	III	28 (40.6)
	IV	40 (58.0)
Histology	Adenocarcinoma	31 (44.9)
	Squamous	15 (21.7)
	Small cell lung cancer	19 (27.6)
	Other	4 (5.8)
Acquisition time of specimens	Before 1st-line treatment	69 (100)
	During treatment	0 (0)

**Table 2:** Clinical features and PD-L1 expression levels in ICI-treated patients.

Age	Sex	Stage	Histology	PD-L1 expression		ICI	Treatment line	Response	PFS, days	Median PFS (95% CI), days
				EBUS-TBNA	TBB					
69	M	IVB	Ad	≥50%	≥50%	Nivolumab	5^th^	PR	917	249 (44‒NR)
62	M	IVB	Ad	≥50%	≥50%	Pembrolizumab	1^st^	PR	249	
38	F	IIIC	Ad	≥50%	≥50%	Atezolizumab	2^nd^	PR	193	
68	M	IVA	Ad	≥50%	≥50%	Pembrolizumab	2^nd^	PD	44	
71	M	IVA	Ad	1‒49%	1‒49%	Pembrolizumab	1^st^	PR	221	50 (20‒NR)
68	M	IIIB	Sq	1‒49%	1‒49%	Pembrolizumab	2^nd^	PD	50	
72	F	IVB	Sq	1‒49%	1‒49%	Pembrolizumab	2^nd^	PD	20	
64	M	IIIA	Ad	<1%	1‒49%	Nivolumab	2^nd^	SD	125	
77	F	IVB	Ad	<1%	1‒49%	Pembrolizumab	3^rd^	SD	85	85 (19‒NR)
66	M	IIIC	Sq	<1%	1‒49%	Atezolizumab	5^th^	PD	19	
78	M	IVB	Ad	<1%	<1%	Pembrolizumab	1^st^	PR	161	
77	F	IVB	Ad	<1%	<1%	Pembrolizumab	1^st^	SD	131	131 (117‒NR)
61	M	IVB	Ad	<1%	<1%	Nivolumab	3^rd^	SD	117	

Abbreviations: PD-L1: programmed cell death ligand 1; ICI: immune checkpoint inhibitor; EBUS-TBNA: endobronchial ultrasound-guided transbronchial needle aspiration; TBB: transbronchial biopsy; PFS: progression-free survival; CI: confidence interval; Ad: adenocarcinoma; PR: partial response; NR: not reached; PD: progressive disease; Sq: squamous cell carcinoma; SD: stable disease.

**Table 3:** Summary of the published studies on the correlation of the expression of PD-L1 between EBUS-TBNA and other histological specimens.

Ref.	Pts, n	Histology	Stage	Specimens used in the study	EBUS-TBNA, n	PD-L1 clone	Comparison	Concordance	ICI efficacy
10	79	45 Ad23 Sq11 NOS	37 stage I18 stage II24 stage III	79 Small biopsy specimens 59 TBB 12 TBNA 8 CTG-CN 79 Resected specimens	12	4059	Resected specimens vs. Matched small biopsies	92.4%	No significant correlation between survival and PD-L1 positivity.
11	160	127 Ad33 Sq	27 stage I40 stage II93 stage III	160 Small biopsy specimens 110 TBB 12 TBNA 38 CTG-CN160 Resected specimens	12	SP142	Resected specimens vs Matched small biopsies	52%	NA
12	97	53 Ad17 Sq27 NEC	NA	97 EBUS-TBNA20 TBB11 Resected specimens 6 Lungs 5 LN	97	EPR1161	EBUS-TBNA vs (1) Matched TBB (2) Matched resected lung (3) Matched resected LN	(1) r = 0.75 (2) r = 0.75 (3) r = 0.93	NA
13	188	141 Ad32 Sq6 NOS9 Others	NA	174 Histology specimens77 Resected lung 25 Resected other sites 39 CTG-CN 15 TBB 18 Other biopsies40 Cytology specimens 25 EBUS-TBNA 3 Other FNA 12 Effusions	25	22C3	Resected specimens vs. Matched small biopsy specimens	91%	NA
14	61	39 Ad21 Sq1 Others	2 stage I17 stage II41 stage III1 stage IV	61 EBUS-TBNA61 Resected specimens	61	22C3	Resected specimens vs. Matched EBUS-TBNA	87% (TPS ≥1%)82% (TPS ≥50%)	NA
15	153	91 Ad 55 Sq 7 Others	48 stage I, II 92 stage III, IV 13 NA	153 Small biopsy specimens 110 TBB 23 TBNA 20 CTG- or USG-CN 30 Resected specimens	23	22C3	Resected specimens vs. Matched small biopsy specimens	86.7%	NA
16	577	378 Ad151 Sq48 Others	307 stage IV270 NA	577 Small biopsy specimens189 EBUS-TBNA/EUS-FNA 72 TBB 167 CTG-CN 6 Pleural124 Resected specimens19 Other site specimens	189	22C3	None	None	11/56 (19.6%) achieved PR. All responders had high PD-L1 expression.
17	120	94 Ad17 Sq7 NOS2 Others	1 stage I 7 stage II 50 stage III 58 stage IV	120 EBUS-TBNA 18 Matched histologic specimens 1 TBB 11 Resected specimens 1 Pleural biopsy 4 Core biopsies 1 Autopsy	120	22C3	EBUS-TBNA vs. Matched histologic specimens	78%	NA
18	71	39 Ad24 Sq8 Others	4 stage II 22 stage III 45 stage IV	71 Small biopsy specimens 71 EBUS-TBNA 68 Matched TBB 28 Resected specimens 13 Lungs 15 Metastases	71	E1L3N	EBUS-TBNA vs. (1) TBB (2) Resected lung (3) Resected metastases.	(1) κ = 0.63 (2) κ = 0.68 (3) κ = 1.0	NA

Abbreviations: PD-L1: programmed cell death ligand 1; EBUS-TBNA: endobronchial ultrasound-guided transbronchial needle aspiration; ICI: immune checkpoint inhibitor; Ad: adenocarcinoma; Sq: squamous cell carcinoma; NOS: not otherwise specified; TBB: transbronchial biopsy; CTG-CN: computed tomography-guided core needle biopsy; NA: not available; NEC: neuroendocrine carcinoma; LN: lymph node; FNA: fine needle aspiration; TPS: tumor proportion score; USG-CN: ultrasound-guided core needle biopsy; PR: partial response.

## Abbreviations

CI: confidence interval
EBUS-TBNA: endobronchial ultrasound-guided transbronchial needle aspiration
ICI: immune checkpoint inhibitor
IHC: immunohistochemistry
NSCLC: non-small cell lung cancer
PD-1: programmed death protein-1
PD-L1: programmed death ligand 1
PFS: progression-free survival
SCLC: small cell lung cancer
TBB: transbronchial biopsy
TPS: tumor proportion score
